# Spinal Cord Perfusion Pressure After Resection of Intramedullary Spinal Cord Tumors

**DOI:** 10.7759/cureus.110475

**Published:** 2026-06-08

**Authors:** John Paul G Kolcun, Ryan Kelly, John Otoole

**Affiliations:** 1 Neurological Surgery, Rush University Medical Center, Chicago, USA; 2 Neurosurgery, Rush University Medical Center, Chicago, USA

**Keywords:** intramedullary tumors, intrathecal pressure, mean arterial pressure, spinal cord perfusion pressure, spine

## Abstract

Introduction

Surgical resection of intramedullary spinal cord tumors (IMSCTs) involves direct, albeit controlled, trauma to spinal cord tissue. Postoperative management after resection of IMSCTs can include elevated mean arterial pressure (MAP) to augment blood flow to the injured cord. We measured postoperative spinal cord perfusion pressure (SCPP) in five consecutive IMSCT cases and reviewed postoperative hemodynamic management in a continuous, single-surgeon series.

Methods

This study was conducted at Rush University Medical Center, Chicago, Illinois. All IMSCT patients who underwent surgical resection by the senior author over a 15-year period were reviewed. We reviewed all practices regarding postoperative MAP goals, vasopressor administration, and intensive care unit (ICU) stay. In five consecutive cases, we transduced intrathecal pressure (ITP) via lumbar drain (LD) to calculate SCPP (SCPP = MAP - ITP).

Results

Thirty-eight patients were reviewed. Twenty-nine patients were managed with postoperative MAP goals (76.3%) for 3.0 ± 1.4 days, with 2.5 ± 1.7 days in the ICU. Twelve patients required pressor administration (31.6%) for 0.9 ± 1.5 days. All patients with SCPP measurement had elevated MAP goals postoperatively, and 4 of 5 required pressor administration. Daily average ITP was normal for the duration of measurement; the highest ITP was measured postoperative days 0 and 1. All patients maintained normal (and significantly elevated) SCPP.

Conclusions

SCPP is maintained after resection of IMSCT tumors, primarily due to low ITP. Postoperative elevated MAP goals may be unnecessary, so long as normotension is maintained. Future prospective studies with correlated clinical outcomes are needed to confirm this principle.

## Introduction

Primary spinal tumors are a rare pathology, representing only 2-4% of all central nervous system neoplasms [1]. These tumors occur throughout the spine, including the extradural, intradural extramedullary, and intradural intramedullary compartments. Intramedullary spinal cord tumors (IMSCTs) are the least prevalent, comprising only 20-30% of all spinal intradural neoplasms [1,2]. Treatment of IMSCTs is challenging due to their intimate proximity to normal nervous tissue and thus the inherent risk of neurological injury during surgical access and resection. Therefore, the benefit of resection must be weighed against the expected postoperative decline in functional status and associated quality of life [3]. In this way, surgical trauma to spinal cord tissue during IMSCT surgery can be considered an “iatrogenic spinal cord injury” (SCI), and frequently results in additional rehabilitation needs, with associated delays in patient recovery and increased health-related costs for these patients [4,5].

Following the 2013 Congress of Neurological Surgeons Guidelines formal recommendation for elevated mean arterial pressure (MAP) in acute SCI patients, our institution adopted routine MAP goals for all IMSCT patients in the immediate postoperative period [6]. However, since the publication of those guidelines, an increasing body of evidence has supported spinal cord perfusion pressure (SCPP) as a more sensitive predictor of neurologic recovery after SCI, particularly in the acute and early subacute periods [7]. Specifically, maintaining a SCPP >50mmHg has been associated with superior odds of significant neurologic improvement at six months after SCI [8].

With these considerations, we sought to examine the utility of postoperative MAP goals and determine any relevant changes to SCPP after IMSCT resection. We retrospectively reviewed our consecutive series of IMSCT cases to evaluate the use of MAP goals and their associated impact on hospital course (e.g., intensive care unit (ICU) stay, pressor consumption). Furthermore, we measured actual postoperative SCPP in a small series of recent consecutive IMSCT cases.

## Materials and methods

This study was conducted at Rush University Medical Center, Chicago, Illinois. We reviewed all IMSCT tumor cases performed by the senior author over a 15-year period, including relevant demographics, details of clinical presentation, imaging findings, surgical treatment and hospital course, and final pathology. Inclusion criteria consisted of those with solitary intramedullary spine tumors who underwent surgical resection. Exclusion criteria included those with underlying cardiovascular pathology that would have made it unsafe to maintain desired MAP goals, those without intramedullary tumors, and patients aged <18 years. Specifically, we tracked practice changes regarding MAP management in the perioperative period. In recent cases that underwent SCPP monitoring, we also reviewed MAP, intrathecal pressure (ITP), and SCPP measurements in the immediate perioperative period.

SCPP is defined as the difference between MAP and ITP (SCPP = MAP - ITP). ITP was transduced using a lumbar drain (LD), which was placed intraoperatively (under anesthesia, immediately before surgery commenced). “Normal” ITP can vary widely, and has been described between 6-25cmH2O, with an average of 18cmH2O in healthy adults [9]. Our recent decision to incorporate LD placement during surgery for IMSCT patients was based on the evidence described above for SCPP as a superior measure for BP management in SCI patients. We measured opening pressure (OP) in all cases. The length of LD catheter insertion was tailored to the operative segment (e.g., a shorter insertion depth was used for a low thoracic tumor). Postoperatively, ITP was transduced each hour, with the patient lying flat, and the transducer levelled at the heart. In order to avoid immediate changes to ICU protocols, no alterations to BP management were made for these initial patients in response to ITP/SCPP measurements. Rather, these values were recorded, while MAP goals were managed according to our standard clinical practice. This included maintaining MAP between >85-90 mmHg through the use of vasopressor agents with an arterial line initially placed intraoperatively. Measurements were recorded over the course of the patient's ICU stay, which typically lasted three days.

Continuous variables are reported as mean±standard deviation; categorical variables, as N (%). This study was approved by the Institutional Review Board (IRB) of Rush University Medical Center (no. 23051105-IRB01), and patient consent was obtained prior to surgical intervention. The authors have no conflicts of interest to disclose.

## Results

We identified 38 patients who met the criteria for inclusion. Patients were 50.1 ± 13.4 years old, 52.6% female, and included four current and 10 former smokers. Patients’ McCormick grade at presentation was 2.4 ± 0.7; specifically, the most common presenting symptom was numbness (76.3%), followed by motor weakness (68.4%), generalized myelopathy (63.2%), and altered gait (60.5%) (Table 1). Most tumors were located in the thoracic spine (65.8%). On average, tumors spanned 34.1 ± 28.8mm in length and 9.2 ± 2.8mm in diameter. At the level of pathology, the patients’ spinal canals measured 13.8±2.6mm in diameter (Table 2).

**Table 1:** Demographics, medical comorbidities, and clinical presentation BMI: body-mass index; HTN: hypertension; DM: diabetes mellitus; PVD/CAD: peripheral vascular disease/coronary artery disease; sx: symptoms. Continuous variables shown as mean, ±standard deviation; categorical variables shown as N, %.

Age	50.1	±13.4
Female	20	52.6%
BMI	30.9	±6.9
Current smoker	4	10.5%
Former smoker	10	26.3%
HTN	15	39.5%
DM	3	7.9%
PVD/CAD	2	5.3%
McCormick grade	2.4	±0.7
Numbness	29	76.3%
Weakness	26	68.4%
Radiculopathy	10	26.3%
Myelopathy	24	63.2%
Axial pain	11	29%
Gait disturbance	23	60.5%
Bowel/bladder	11	29%
Other/non-specific sx	1	2.6%

**Table 2:** Tumor characteristics Continuous variables shown as mean, ±standard deviation; categorical variables shown as N, %

Cervical region	17	44.7%
Thoracic region	25	65.8%
Lumbar region	3	7.9%
Tumor length (mm)	34.1	±28.8
Tumor diameter (mm)	9.2	±2.8
Local canal diameter (mm)	13.8	±2.6

Most patients were treated by laminectomy for resection (68.4%), with the remainder undergoing laminoplasty (31.6%). There were no operative complications. The most common pathology was ependymoma (16, 42.1%). Among all cases, 29 patients were managed with postoperative MAP goals, typically >85-90 mmHg (76.3%), which were maintained for 3.0 ± 1.4 days, comprising a 2.5 ± 1.7 day ICU stay. Twelve patients required intravenous pressor usage to meet MAP goals (31.6%), for an average of 0.9 ± 1.5 days. All cases without postoperative MAP goals were performed prior to 2013. The patients’ average McCormick grades were 2.3 ± 0.9 at the final follow-up (Table 3).

**Table 3:** Surgical approach and treatment course MAP: mean arterial pressure; ICU: intensive care unit; LOS: length of stay. Continuous variables shown as mean, ±standard deviation; categorical variables shown as N, %.

Laminectomy	26	68.4%
Laminoplasty	12	31.6%
Estimated blood loss (cc)	159.5	±154.8
Surgical complications	0	0%
Postoperative MAP goals	29	76.3%
No. MAP goal days	3	±1.4
Pressor usage	12	31.6%
No. pressor days	0.9	±1.5
ICU LOS	2.5	±1.7
Perioperative complication	7	1.8%
Final McCormick grade	2.3	±0.9

**Figure 1:**
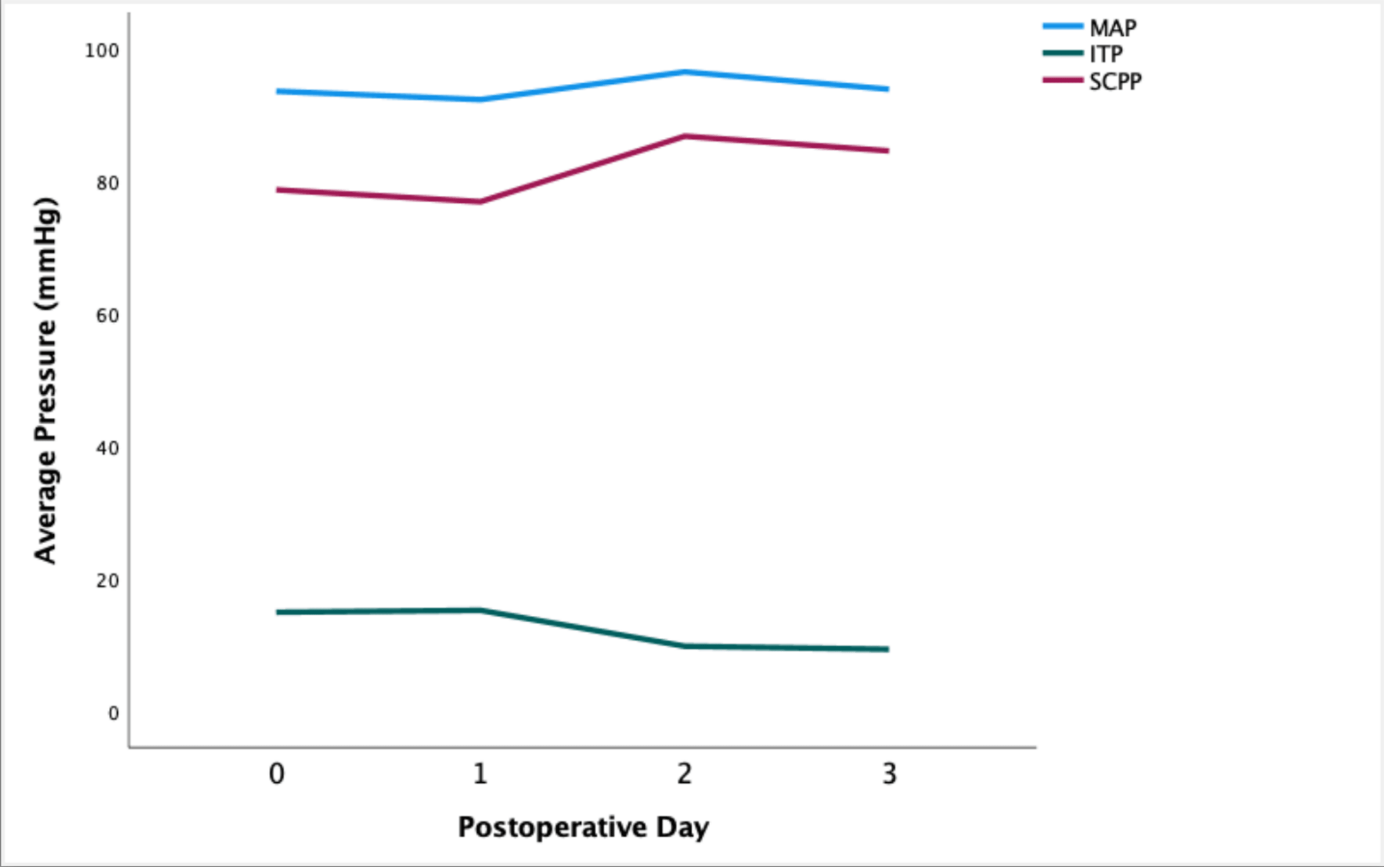
Average daily parameters of IMSCT patients undergoing postoperative SCPP monitoring MAP: mean arterial pressure; ITP: intrathecal pressure; SCPP: spinal cord perfusion pressure

Among the five patients who underwent SCPP monitoring, opening pressure at the time of LD placement was 14.4 ± 2.6 cm H_2_O. All five patients had elevated MAP goals postoperatively, and four of five required pressor administration to meet these goals. The daily average ITP was normal for all five patients (normal average 18 cmH_2_O). The highest ITP values were measured on postoperative days 0 and 1 (14.9 ± 10.0 and 15.7 ± 8.3, respectively), with lower average values on postoperative days 2 and 3 (9.9 ± 7.1 and 8.3 ± 6.1, respectively). All patients maintained acceptable SCPP throughout the monitoring period (SCPP >50 each day) (Table 4, Figure 1).

**Table 4:** Average measured pressure parameters in SCPP patients POD: postoperative day; MAP: mean arterial pressure; ITP: intrathecal pressure; SCPP: spinal cord perfusion pressure. Continuous variables shown as mean, ±standard deviation.

POD 0		
Opening pressure	14.4	±2.6
MAP	93.5	±9.5
ITP	14.9	±10
SCPP	78.7	±10.4
POD 1		
MAP	92.4	±11.8
ITP	15.7	±8.3
SCPP	76.9	±13.7
POD 2		
MAP	96.6	±10.3
ITP	9.9	±7.1
SCPP	86.8	±9.1
POD 3		
MAP	93.6	±9.7
ITP	8.3	±6.1
SCPP	84.6	±10.3

LD function and ITP transduction were monitored personally by the authors each day postoperatively. In the early postoperative period, the transducer generated a flat waveform with minimal variation during systole or respiration; however, the LD reliably exhibited anterograde/retrograde flow, confirming functionality. In all five cases, the transduced waveform began to show systolic peaks and reliable, expected respiratory variation by the morning of postoperative day 2.

## Discussion

This “proof of concept” study demonstrates that SCPP may be maintained in the postoperative period after resection of IMSCTs, seemingly regardless of elevated MAP goals. To our knowledge, this represents the first investigation of perioperative SCPP in this patient population.

We conclude that SCPP maintenance after IMSCT resection is driven by a significantly decreased postoperative ITP. First, it is physically intuitive that a reduction in intrathecal contents (i.e., tumor volume, significant CSF loss) would reduce ITP. Second, measured ITP - even immediately postoperatively - would yield a SCPP >50 without MAP elevation, so long as normotension is maintained. Finally, we observed minimal waveforms on LD transduction during postoperative days 0-1. We interpret this to reflect an intrathecal CSF volume so reduced as to poorly transmit the pressure wave of systole. Thus, actual ITP on postoperative days 0 and 1 were likely significantly lower than the transduced values we recorded. The emergence of systolic peaks and respiratory variation on the transduced waveform (postoperative day 2) coincided with a significant drop in daily average ITP; again suggesting that actual postoperative ITP was lower than measured values in the early postoperative period, as would be expected. In the setting of decreased medullary perfusion, increasing the MAP can lead to improved spinal cord perfusion and ideally the reduction of ischemic changes within the spinal cord.

At our institution, this conclusion has meaningful implications for our standard practice in these patients’ postoperative management. In our series, patients generally spent 2-3 days in the ICU and 1-2 days on pressors postoperatively. While commonly prescribed, vasopressor administration is not without risk [10]. Furthermore, extended ICU stays are associated with a significant increase in health care-related costs [11]. Our present findings suggest that a prolonged ICU stay for hemodynamic management with vasopressor administration may not be necessary after uncomplicated IMSCT resection, thus avoiding the associated costs and risks to these patients, so long as routine normotension is maintained in the postoperative period.

SCPP is a biologically plausible concept whose utility in SCI management is supported by preclinical animal models and prospective human-subject evidence [7, 12]. A recent multi-center randomized controlled trial assessing the use of SCPP in patients with acute cervical SCI demonstrated that those with MAP elevation and CSF drainage demonstrated a lower ITP (5.3 ± 2.5 mmHg) compared to the control group that received MAP elevation (15 ± 3.0 mmHg) [13]. While this and other studies are limited in their small sizes, their application to IMSCT resection can be inferred. However, SCPP physiology can be rationally considered in the early postoperative management of this population, given the fact that spinal cord tissue is by definition “injured” during IMSCT resection, but viable tissue surrounding the resection cavity may benefit from augmented blood flow during the acute postoperative phase. In addition to blood delivery, the SCPP model incorporates ITP as a “barrier to entry” for actual perfusion to the injured cord.

The SCI to IMSCT analogy is limited in two critical aspects. First, the nature of injury: a closed blunt SCI will result in greater cord edema than a controlled penetrating surgical “injury.” Second, the dramatic volumetric reduction of intrathecal contents during IMSCT resection likely offsets reactive postoperative cord swelling. The chronic, intrinsic, and possibly infiltrative relationship between IMSCT neoplasms and native cord tissue must also be considered. It had previously been demonstrated that an increase in ITP following acute SCI was maintained in the postoperative period and that drainage of CSF during this time period decreases ITP [14]. All SCPP patients had normal OP at the time of LD placement, although these measurements could be confounded by general anesthesia. However, despite the expansive nature of IMSCT lesions (as opposed to extrinsic spinal cord compression), there was no significant stenosis at the level of the tumor in our series (Table 3). Therefore, a normal baseline ITP would be expected. However, variations in tissue density/compliance, microvasculature proliferation and flow dynamics, and variable metabolic demands likely all impact regional cord perfusion in ways that, while reasonable to consider, may not yet be fully understood [15].

Limitations

This is a single-center, single-surgeon small case series, which limits the generalizability of our findings. Additionally, not all surgeons may utilize postoperative MAP goals after IMSCT resection, and therefore, the principle of SCPP may not be relevant for practice in such a setting. Nevertheless, for the small group of patients in this study, hourly measurements of ITP for up to three days across these subjects were remarkably consistent. Furthermore, our results (low ITP with preserved SCPP) validate intuitive physiological expectations in this setting. The present study establishes a baseline for future investigations into perioperative spinal cord perfusion in the setting of IMSCTs.

## Conclusions

SCPP appears to be reliably maintained following resection of IMSCTs, most likely due to a postoperative reduction in intrathecal pressure rather than dependence on supraphysiologic mean arterial pressure. Our findings suggest that, in uncomplicated cases, routine elevation of MAP with vasopressor support may offer limited physiological benefit when normotension alone is sufficient to preserve adequate spinal cord perfusion. Given the well-established risks associated with vasopressor therapy and prolonged ICU admission, these results raise important questions regarding the necessity and value of aggressive postoperative hemodynamic protocols in this patient population. Although limited by sample size and retrospective design, this study provides foundational evidence supporting a more individualized approach to postoperative blood pressure management after IMSCT resection. Larger prospective studies incorporating neurological outcomes are warranted to validate these findings and refine postoperative management strategies aimed at optimizing patient safety, resource utilization, and recovery.
